# Prevalent Health Problems among Nepalese Underground Construction Workers

**DOI:** 10.1155/2020/9436068

**Published:** 2020-12-07

**Authors:** Rajan Ghimire, Ganesh Prasad Neupane

**Affiliations:** ^1^Clinical Coordinator, General Practice and Emergency Medicine, District Hospital, Terhathum, Nepal; ^2^Annapurna Neurological Institute and Allied Sciences, Maitighar Mandala, Kathmandu, Nepal; ^3^Environment and Public Relation Section, Upper Tamakoshi Hydroelectric Project, Dolakha, Nepal

## Abstract

**Background:**

Workplace is associated with exposure to various products, which can be associated with adverse health outcomes. It is true with underground construction work. This study calculated the prevalence of common health problems among Nepalese underground construction workers in comparison to heavy construction workers. This type of study is rare in the context of Nepal and other developing countries, and we hope that the findings will help to take precautions for the prevention of these conditions.

**Method:**

It was a retrospective study based on the clinical record of outpatient cases and general health checkups of all Nepalese workers available at the Project clinic, Upper Tamakoshi Hydroelectric Project, Gongar, Bigu, Dolakha. We studied three hundred and ninety-eight workers. We used multipurpose analysis and conducted the Chi-square test and calculated correlations and odds ratios.

**Results:**

Two hundred and sixteen (54.3%) participants worked inside the tunnel, and 182 (43.2%) participants worked outside the tunnel. Respiratory disease, mainly upper respiratory tract infection (URTI) (23.4%), is the most common presentation among construction workers followed by injuries (16.7%). Injuries and hypertension were significantly higher in inside the tunnel workers, and acute gastroenteritis was significantly (*p* value <0.05) higher in outside the tunnel workers. Increasing age increased the chance of hypertension and cutaneous fungal infection among construction workers. Further studies are required for the analysis of risk factors associated with these health conditions.

**Conclusion:**

Respiratory problems are the most common health problem in underground construction workers; however, injuries and hypertension were significantly higher in tunnel workers. Acute gastroenteritis was significantly higher among outside the tunnel workers. Workplace safety should be the priority of every construction site, especially focusing to prevent respiratory problems, injuries, and accidents.

## 1. Introduction

In the current situation, underground construction is increasing day by day in various forms such as underground railways, mines, underground settlements, and tunnels for various purposes [1]. As every sort of construction work poses health risk in some way or the other, underground construction is not also totally free of health hazards. Underground construction sites have an increased amount of dust particles including silica and quartz. Diesel-powered machinery and wielding works add up gaseous particulate to the underground working sites. Blasting and rock drilling works not only increase dust particles but also produce sound, which is not safe to the human ear. Besides the risks in the work place, living conditions provided to these workers can also be a source for many diseases.

In comparison to the general workforce, construction workers experience a high risk of disability from respiratory diseases, musculoskeletal diseases, and injuries [2]. Underground construction workers have an increased risk of obstructive lung disease [3] and decreased lung function due to long-term exposure to nitrogen dioxide and dust [2].

The main aim of this study is to calculate the prevalence of common health problems (diseases) among underground workers in comparison to heavy-construction workers. Different geography and working conditions lead to difference in disease occurrence. In comparison to developed nations, developing nations lack standard safety measures in the work place including personal protective equipment.

### 1.1. Research Gap

Underground construction work is increasing in Nepalese context. These works can be for development of hydroelectric projects, road construction, or underground settlement. We lack data for diseases or other health issues associated with underground construction work in Nepal. This sort of study has been conducted for the first time in the Nepalese context. So, this type of study will help to identify health problems associated with underground work. It will further help to conduct risk assessment studies for a particular disease and to enforce risk reduction measures for the policymakers, as well as electric power project developers.

### 1.2. Literature Review

Vibration from the driller machine can lead to prolonged neuropathy which manifests as a hand-vibration syndrome [4, 5]. They share common kitchen and dormitory and are subjected to various communicable diseases such as diarrhea, dysentery, and typhoid [6]. Tunnel workers are prone to different respiratory problems [7, 8], injuries, musculoskeletal pain, dermatitis, and communicable disease [6]. Many machines involved in underground construction work are powered by mineral oils (diesel), as well as by electric current. A considerable amount of diesel exhausts are emitted by the use of automobiles, in addition to other pollutants such as gases and dust from blasting [3]. Diesel exhaust causes an increase in neutrophils in airway lavage, together with an adverse influence on the phagocytosis by alveolar macrophages in vitro [9]. Also, diesel particulate matter increases the risk of lung cancer [8].

These workers face high temperatures, high humidity, low oxygen level, high concentration of carbon dioxide, nitrogen gases, low natural light exposure, and other physical stresses related to underground work [10]. Underground particulate matter may be more toxic than exposure to ambient/urban particulate matter, especially in terms of endpoints related to reactive oxygen species generation and oxidative stress. This appears to be predominantly a result of the metal-rich nature of underground particulate matter, which is suggestive of increased health risks [11]. Most of these workers are separated from their family members. All these factors lead to the development of physical discomfort and psychological stress and illness [12].

## 2. Methodology

This study was a cross-sectional study based on the clinical record of outpatient cases and general health checkups of all Nepalese workers available at the Project clinic, Upper Tamakoshi Hydroelectric Project, Gongar, Bigu rural municipality, Dolakha, Nepal. We collected data of one year from August 2013 to July 2014.

Most of the project construction activities are underground. The powerhouse and transformer cavern is located 1200 m from the portal site. Out of the total 19 km underground works, 8.0 km is the headrace tunnel and 2.98 km is a tailrace tunnel and has many audits at various locations and 340 m road tunnel. Outside of the tunnel, workers work in offices, mechanical workshops, rock crushers, and kitchen. Contractors allocated the place of work during the time of the job contract. Inside the tunnel, there were adequate amount of artificial lights, ventilation pipes connected to the external environment, and fans. There was a provision to check the amount of dust particles, the amount of harmful gases, and temperature inside the tunnel regularly. Consultant office and contractors jointly took actions to bring them to the recommended level. Contractors provided mask, safety helmet, gloves, and boot as personal protective equipment to workers. In the tunnel, works such as drilling the rock, blasting, concreting the rocks, and wielding of steel frameworks for rock support were conducted. Machines were diesel powered. Workers had two shifts in a day, twelve hours each.

### 2.1. Data Collection

We collected data using a self-developed format to enter demographic data, place of work, use of tobacco products, and recognized health problems in Microsoft office excel. We used the record of general health checkup and an outpatient visit to collect data. General health check was performed during the recruitment of workers, and if they had any health issues during work period, they were consulted in the outpatient department. As we collected, secondary data disease/diagnosis were recorded as per the availability in the record book. Only Nepalese workers who visited the project clinic were included in the study. Patients who visited the clinic for the same problem within 21 days and patients with incomplete data were excluded from the study.

### 2.2. Statistical Analysis

First, data was entered in Microsoft Excel 2016 pack. A single person had multiple health issues at the same visit or any time during the study period, so the multipurpose analysis was performed in IBM SPSS 25.0 version. We used the Chi-square test, correlation, and odds ratio to find the association of health problems with the place of work. A *p* value ≤0.05 was considered statistically significant. Bivariate logistic regression analysis was conducted to analyze the association of confounding factors, namely, age, sex, and smoking, with the disease.

## 3. Results

Out of 398 participants, 216 (54.3%) worked in the tunnel/underground construction work and 182 (45.7%) worked outside the tunnel. Data for age were normally distributed, and they were negatively skewed for all the diseases. The overall mean age was 29.29 years (SD = 9.018; skewness = 0.858). The mean age was 30 years (SD = 10 years) in tunnel workers and 29 years (SD = 8 years) in participants working outside the tunnel. Out of 398 participants, 377 (94.7%) were male while 21 (5.3%) were female. Among them, 216 (57.3%) males worked inside the tunnel while 161 (42.7%) worked outside the tunnel and all females worked outside the tunnel. Forty-three (10.8%) participants chewed tobacco and one hundred and fifty (37.69%) participants smoked tobacco with a mean of 1.92 pack-year (SD = 4.88). Tunnel workers smoked 2.64 pack-year (SD = 5.53) of cigarettes on average, and outside tunnel workers smoked an average of 1.05 pack-year (SD = 3.81) of cigarettes.

Respiratory disease, mainly upper respiratory tract infection (URTI) (23.4%), is the most common presentation among all the construction workers followed by injuries (16.7%) (Table 1). Thirty-two (6.7%) participants had no health issues. Other health problems include otitis media, neuropathy, urinary tract infection, arthritis, anxiety disorder, herpes zoster, hemorrhoids, dental caries, abscess, conjunctivitis, cellulitis, sexually transmitted infection, and limb bone fracture.

Upper respiratory tract infection is the most common problem among workers inside the tunnel but has no significant difference. Injuries and hypertension were significantly higher among tunnel workers, and acute gastroenteritis was significantly higher among the workers outside the tunnel (Table 2). Twenty-one workers (9.7%) in inside tunnel workers and 11 workers (6.0%) in outside tunnel workers had no health issues.

There was a significant association of age with hypertension and cutaneous fungal infection (Table 3). Mean age for patients with and without hypertension was 34 and 29 years; and for cutaneous fungal infection, it was 26 and 29 years. Increasing age increased the risk of hypertension and cutaneous fungal infection.

## 4. Discussion

Construction workers are exposed to different environmental pollutants including dust, gases, welding fumes, and diesel particulate matters. Most of the machines were diesel powered. Normal cotton masks were provided to workers. Besides these, they used a common residential dormitory that leads to an increase in the prevalence of communicable diseases such as respiratory problems and gastroenteritis. As most of the workers stay away from the family, they are prone to develop some psychiatric illness and hypertension. Noise, dust, high temperature, high humidity, vibration, and poor lighting impose a risk to the health of mine workers [6].

Workers working in the tunnel are exposed to explosives and their fumes which reduce their lung function and cause respiratory disease [3, 13–15]. Compared with the reference subjects, the tunnel workers had a significant decrease in predicted forced vital capacity (FVC) % and forced expiratory volume in one second (FEV1) % when related to years of exposure [3, 16]. The annual decrease in FEV1 in tunnel construction workers was 20–31 ml, higher than the low exposed workers depending on job group for both nonsmokers and ever smokers [10]. In tunnel workers, with occupational quartz exposure and normal chest radiographs, the duration of occupational quartz exposure was an independent predictor for spirometric airflow limitation [17, 18], and these workers had various respiratory symptoms despite the level of respirable dust below the recommended level [7]. Tunnel workers with pneumoconiosis and COPD had excess mortality [19]. Significantly elevated mortality from all causes, cardiovascular disease, nonmalignant respiratory disease, and lung cancer, were observed among silica-exposed workers while elevated mortality from nonmalignant respiratory disease and lung cancer was observed among smokers [20].

Our study showed that respiratory disease, mainly upper respiratory tract infection (URTI) (23.4%), was the most common presentation in construction workers; however, there was no significant difference between respiratory problems in tunnel workers and outside tunnel workers. Workers working outside the tunnel were also exposed to environmental pollutants such as dust, diesel fumes of the garage, and silica/other minerals from stone grinders, which might have led to a significant number of respiratory problems among workers in the outside tunnel group too. All these facts point towards workplace safety. Exposure to blasting fumes and diesel exhaust emissions should be reduced, and respiratory safety devices should be used to protect workers against dust, diesel particulate matter, noxious gases, and nitrogen dioxide exposure [10].

We found that 16.7% of construction workers had injuries, which was significantly higher than in outside tunnel workers. In another study, major causes of disability were musculoskeletal (45%) and cardiovascular diseases (19%). Fatal injuries due to falls and being struck by falling objects pose particular health hazards among construction workers [21]. In comparison with the general workforce, construction workers experienced a higher risk of disability from cancer, respiratory diseases, musculoskeletal diseases, injuries/poisoning, and all causes combined [2]. Drilling machine operators, welders, flame cutters, reinforcing-iron workers, and heavy-equipment operators had the highest proportionate mortality ratios [22]. The associations of occupational stress with physical, emotional, and social life and with limitation of day-to-day activities among tunnel workers were found to be statistically significant [23].

In our study, 6.1% of all workers had a cutaneous fungal infection and 4.4% had dermatitis. In another study, the prevalence rates of occupational skin disease were 1.35% in all workers and 62.2% in workers with skin diseases. The prevalence of skin disease in construction workers was 1.6% [24]. In another group of construction workers, they had frictional callosities in the palm (*n* = 18, 19.6%), contact dermatitis (*n* = 4, 4.3%), dry, fissured and scaly skin, infectious skin lesion, tinea cruris, and ulcers on hands and/or soles [25].

Underground workers had various phobias that strengthen their discomfort and increase mental health problems [12]. Nature of tasks, lack of personal and family time, increase in the cost of living, and fears about job security act as powerful stressors. Coping strategies including acceptance, self-blame, substance abuse, and disengagement are associated with higher levels of psychological distress [26]. Another study identified missing special events (86%), relationship problems with partners (68%), financial stress (62%), shift rosters (62%), and social isolation (60%) as stressors among mining and construction workers [27].

### 4.1. Limitations

In our study, few workers might already have health issues not detected before starting work. They might have been already exposed to pollutants, either environmental or from previous workplaces. As the study was based on secondary data, strict disease definitions could not be made and, hence, followed. All disease conditions were diagnosed based on history and clinical examinations only. There was no laboratory support.

### 4.2. Recommendations and Study Implications

This study tried to identify the common health issues among underground construction workers in Nepal. Based on the findings, we suggest the proper work place safety to decrease injuries. We suggest the proper use of personal protective equipment for the construction workers to decrease incidence of respiratory and dermatological problems. Furthermore, a well-designed prospective study is required to establish the association of underground construction work with identified common health problems along with the risk factors.

## 5. Conclusions

We conducted a retrospective cross-sectional study with an objective to find the common health problems among the Nepalese underground construction workers. Respiratory problems, injuries, hypertension, and skin conditions were common among construction workers with significant differences seen with injuries and hypertension. The gastrointestinal problem was significantly higher among outside tunnel worker groups. Increasing age was found to be associated with hypertension and cutaneous fungal infection among tunnel workers. Workplace safety should be the priority of every construction work, especially focusing to prevent respiratory problems, injuries, and accidents. Further prospective studies are required to find the risk factors for these diseases.

## Figures and Tables

**Table 1:** Common health problems among all construction workers based on multipurpose analysis.

Disease	Frequency of responses	Percentage
URTI	112	23.4
Injuries	80	16.7
APD	30	6.3
Hypertension	39	8.2
Cutaneous fungal infection	29	6.1
Malabsorption	21	4.4
Acute gastroenteritis	26	5.4
Foreign body eye/ear	26	5.4
Dermatitis	21	4.4
Refractive error of eye	34	7.1
Other	28	5.9

**Table 2:** Chi-square test, correlation, and odds ratio analysis (multipurpose analysis) of health problems among the inside tunnel workers and outside tunnel workers.

S.N	Disease	Frequency/percentage within group		Chi-square		Correlation		Odds ratio			
		Inside tunnel	Outside tunnel	Value	*p* value	Value	*p* value	Value	*p* value	95% C.I.	
										Lower	Upper
1	URTI	65/30.1	47/25.8	0.89	0.35	0.047	0.347	1.236	0.346	0.795	1.922
2	Injuries	52/24.1	42/15.4	4.644	**0.031**	0.108	**0.031**	1	**0.032**	1.048	2.902
3	APD	14/46.5	16/8.8	0.756	0.385	−0.044	0.386	0.719	0.386	0.341	1.516
4	Hypertension	30/13.9	9/4.9	8.939	**0.003**	0.15	**0.003**	3.1	**0.004**	1.431	6.717
5	Cutaneous fungal infection	12/5.6	17/9.3	2.095	0.148	−0.073	0.149	0.571	0.152	0.265	1.229
6	Malabsorption	12/5.6	9/4.9	0.074	0.786	0.014	0.787	1.131	0.786	0.465	2.747
7	Acute gastroenteritis	8/3.7	18/9.9	6.191	**0.013**	−0.125	**0.013**	0.350	**0.017**	0.149	0.826
8	Foreign body eye/ear	11/5.1	15/8.2	1.604	0.205	−0.063	0.206	0.597	0.209	0.267	1.335
9	Dermatitis	9/4.2	12/6.6	1.164	0.281	−0.054	0.282	0.616	0.285	0.254	1.497
10	Refractive error of the eye	24/11.1	10/5.5	3.988	**0.046**	0.1	**0.046**	2.15	0.05	1.00	4.624
11	Other	13/6.0	15/8.2	0.746	0.388	−0.043	0.389	0.713	0.389	0.33	1.540

^∗^Bold numbers indicate significant findings with a *p* value ≤0.05.

**Table 3:** Bivariate logistic regression for the association of demographic variables with health problems among all construction workers.

Disease	Age		Sex		Smoking		Tobacco chew	
	Odds ratio	*p* value	Odds ratio	*p* value	Odds ratio	*p* value	Odds ratio	*p* value
URTI	1.002	0.911	1.647	0.289	0.989	0.683	1.056	0.888
Injuries	1.025	0.164	00	0.998	0.96	0.149	1.272	0.578
APD	0.997	0.915	1.514	0.597	1.00	0.994	2.261	0.137
Hypertension	0.935	**0.001**	0.659	0.63	1.018	0.592	0.952	0.926
Cutaneous fungal infection	1.077	**0.026**	0.527	0.543	0.930	0.115	1.703	0.439
Malabsorption	1.092	0.33	1.62	0.543	0.961	0.612	2.786	0.159
Acute gastroenteritis	0.979	0.401	0.626	0.657	1.114	0.179	0.474	0.348
Foreign body eye/ear	0.993	0.790	1.53	0.59	1.115	0.257	1.761	0.337
Dermatitis	0.962	0.156	3.233	0.09	1.146	0.174	0.589	0.515
Refractive error of the eye	0.982	0.442	1.433	0.645	0.998	0.968	2.151	0.136
Other	0.973	0.272	2.251	0.227	1.045	0.407	0.231	0.168

## Data Availability

Data used to support the findings of this study can be obtained upon request to corresponding author.
